# Water extract of *Ampelopsis grossedentata* improves reproductive performance in laying hens by regulating gut microbiota and PI3K/AKT signaling pathway

**DOI:** 10.1016/j.psj.2025.106368

**Published:** 2025-12-31

**Authors:** Yu Xiao, Jie Long, Lu Liu, Zhaojie Wang, Wei Wang, Peng Huang

**Affiliations:** a Hunan Key Laboratory of Traditional Chinese Veterinary Medicine, Hunan Agricultural University, Changsha 410128, Hunan, China; b Yuelushan Laboratory, Changsha, 410000, China; c College of Animal Science and Technology, Hunan Agricultural University, Changsha 410128, Hunan, China; d College of Veterinary Medicine, Hunan Agricultural University, Changsha 410128, Hunan, China

**Keywords:** Laying hen, Water extract of *A. grossedentata*, PI3K/AKT, Reproductive function, Gut microbiota

## Abstract

Reproductive function plays a central role in health but declines with aging. Recent studies have focused on natural flavonoids to mitigate reproductive aging, with the water extract of *Ampelopsis grossedentata* (WEA) showing promise due to its antioxidant, antitumor, antibacterial, and anti-inflammatory properties. However, the specific bioactive constituents and mechanisms of WEA in alleviating reproductive aging remain unclear. This experiment analyzed the active flavonoid components in WEA and predicted the molecular mechanism by which WEA alleviates reproductive system aging through network pharmacology. Furthermore, based on the predicted molecular mechanisms, the effects of WEA on laying hens' production performance, reproductive function, and intestinal health were explored. A total of 288 laying hens (55 weeks old) were assigned to four groups: control and three WEA doses (50, 150, 250 mg/kg). During the experiment, production performance indicators such as egg weight were recorded daily. After the 8-week period, biological samples were collected for analysis. Network pharmacology identified dihydromyricetin, myricetin, and (-)-epicatechin as key active components, primarily affecting the PI3K/AKT signaling pathway. WEA significantly improved egg quality, immune parameters, reproductive organ morphology, intestinal morphology, and serum sex hormone levels, and reduced inflammatory factor levels. WEA improved ovarian apoptosis by regulating the PI3K/AKT/mTOR pathway and alleviated oviduct inflammation by inhibiting the NF-κB pathway. WEA enhanced intestinal anti-inflammatory and antioxidant functions by regulating NF-κB and Nrf2 pathways and increased short-chain fatty acids in the hindgut. WEA altered the intestinal microbiome, particularly reducing the relative abundance of Methylobacterium-Methylorubrum in the foregut and Bacteroides in the hindgut. Correlation analysis revealed that WEA may alleviate oxidative and inflammatory responses by regulating intestinal microbiota, further impacting the PI3K/AKT cascade. In conclusion, WEA improves antioxidant, anti-inflammatory, and reproductive functions in laying hens by regulating the PI3K/AKT pathway and may alleviate oxidative and inflammatory responses by modulating microbiota. This study provides insights into the mechanism of WEA in improving reproductive performance and intestinal regulation.

## Introduction

In commercial laying hen production, the decline in reproductive performance with aging is a major economic challenge. This decline, characterized by reduced egg production and quality, is closely linked to the aging of the reproductive system, which involves oxidative stress, chronic inflammation, and hormonal dysregulation (Liu et al., 2021; Wu et al., 2022). Nutritional intervention using natural compounds with antioxidant and anti-inflammatory properties represents a promising strategy to mitigate this aging process and sustain productivity.

In this context, natural flavonoids have garnered significant attention. Compounds like quercetin have shown potential in improving reproductive health by modulating hormone secretion and enhancing gut health (Jin et al., 2013). *Ampelopsis grossedentata* Hand.-Mazz., commonly known as vine tea and rich in dihydromyricetin and other flavonoids, has demonstrated potent bioactivities (Carneiro et al., 2020; Ma et al., 2020). Its extracts, such as vine tea extract, exhibit robust antioxidant and anti-inflammatory effects in various models (Guo et al., 2019; Zhang et al., 2021), suggesting its potential applicability in combating reproductive aging. However, despite the established bioactivity of vine tea flavonoids, evidence of their specific effects on alleviating reproductive aging in laying hens remains scarce. The active constituents, core targets, and underlying mechanisms are still unknown, limiting its development as a targeted feed additive. Based on these considerations, we hypothesized that supplementation with the water extract of *Ampelopsis grossedentata* **(WEA)**, rich in flavonoids, could alleviate reproductive aging in laying hens by reducing oxidative stress and inflammation, improving intestinal health, and regulating key molecular pathways associated with reproductive function.

Therefore, this study was designed to test this hypothesis by systematically investigating the protective effects and underlying mechanisms of WEA against reproductive aging in aged laying hens. We first employed network pharmacology to predict potential targets and pathways. Subsequently, we conducted an in vivo feeding trial to evaluate the effects of WEA supplementation on production performance, egg quality, reproductive function, and intestinal health. Furthermore, transcriptomic analysis was integrated to explore the molecular mechanisms involved.

## Materials and methods

### Preparation of WEA

*A. grossedentata* was collected from Yongding District, Zhangjiajie City, Hunan Province, China (Batch No. 20231012HAU), and was authenticated by Professor Peng Huang from Hunan Agricultural University. The dried plant material was crushed into coarse powder, soaked in water (1:15, w/v), and decocted twice for 2 hours each time. The combined decoctions were filtered and concentrated under reduced pressure at 80 °C to obtain the water extract of *A. grossedentata*.

### WEA chemical composition identification

WEA sample was freeze-dried using a vacuum freeze-dryer (Scientz-100F). The freeze-dried sample was crushed using a mixer mill (MM 400, Retsch) with a zirconia bead for 1.5 min at 30 Hz. Dissolve 50 mg of lyophilized powder with 1.2 mL 70% methanol solution, vortex 30 seconds every 30 minutes for 6 times in total. Following centrifugation at 12000 rpm for 3 min, the extracts were filtered (SCAA-104, 0.22 μm pore size; ANPEL, Shanghai, China) before UPLC-MS/MS analysis. The sample extracts were analyzed using a UPLC-ESI-MS/MS system (UPLC, ExionLC™AD; MS, Applied Biosystems 4500 Q TRAP). The main components in WEA samples were determined by UPLC-MS/MS technology, and the possible effective ingredients were obtained by using the existing research results in Pubmed and Web of Science databases.

### Network pharmacology

The active components of WEA were imported into the Swiss Target Prediction database to screen for possible targets with probability > 0.1. The disease targets of reproductive system aging were collected by searching the Gene Cards and TTD databases. The targets from these two databases were merged, duplicate targets were removed, and the remaining targets were the reproductive system aging targets we collected and used in the next study. The screened targets and active compounds were entered into Cytoscape software to construct a "component-target" visualization network. The predicted targets were imported into the STRING database to construct a Protein-Protein Interaction Network (**PPI**). To study the biological function of potential targets in reproductive system aging, the Metascape database was used to collect the Gene Ontology network (**GO**) and Kyoto Encyclopedia of Genes and Genomes network (**KEGG**) data. Subsequently, GO and KEGG data were uploaded to the bioinformatics platform for visual analysis.

### Animal experimental design

The animal experiment procedure was approved by the Animal Ethical and Welfare Committee of Hunan Agricultural University. A total of 288 laying hens (55 weeks old) were randomly assigned to one of four dietary groups, with 8 replicates per group, and 9 laying hens per replicate. The laying hens in four treatments were fed the basal diet supplemented with 0 (Control group), 50 (WEA-L group), 150 (WEA-M group), and 250 mg/kg (WEA-H group) of WEA, respectively. WEA addition amount refers to the existing research results of our team (Wang, 2023). The basal diet was formulated to meet the nutrient requirements of the National Research Council (National Research Council, 1994) and Chinese Feeding Standard of Chicken (NY/T33-2004) (Ministry of Agriculture of the People’s Republic of China, 2004), and its ingredient composition and nutrient levels are presented in Table s1. The laying hens were fed twice a day (6:30 and 14:30), and the eggs were collected regularly (15:00) every day. The feeding and management conditions of laying hens in each group were the same. Laying hens were allowed free access to water and were fed under the constant condition of 16 hours of light and 8 hours of darkness. The experimental period was 8 weeks.

### Sample collection

At the end of the 8th week, one laying hen per replicate was randomly selected to be weighed and slaughtered after fasting for 12 hours. Then, 5 mL of blood was collected from the jugular vein and centrifuged at 3000 rpm for 15 minutes at 4°C. The obtained serum samples were stored at -80°C until analysis. The liver, ovary, oviduct, duodenum, jejunum, ileum, cecum, and chyme were quickly removed, and frozen at -80°C for further analysis, respectively. In the present study, eight biological replicates were included per group (n = 8), with one hen from each replicate considered as an independent biological replicate. This sampling strategy was adopted to ensure the independence of experimental units and to avoid pseudo-replication. All hens were of the same strain and age and were maintained under identical management conditions, including diet formulation, housing environment, temperature, humidity, and lighting program. In addition, sample collection time points, tissue sampling locations, and handling procedures were strictly standardized to minimize variability unrelated to dietary treatments.

### Laying performance and organ indexes assay

During the experiment, the production performance, egg weight and number of malformed eggs of each group were recorded daily per replicate. The feed intake was recorded every two weeks and the feed conversion rate was calculated. The organ indices of spleen, liver, ovary and oviduct were calculated.

### Egg quality assay

Egg samples were collected one day before the end of the experiment, and 8 eggs were randomly selected from each experimental group for egg quality testing. Eggshell strength was measured by an egg force (Orka Technology Ltd). Yolk color, albumen height, and Haugh unit were analyzed by an egg analyzer (Orka Technology Ltd). Vernier calipers (Orka Technology Ltd) were used to determine the eggshell thickness, length and width of the egg, yolk height, and yolk diameter.

### Serum biochemical parameters, sex hormones, and inflammatory factors

Serum biochemical parameters including Alanine aminotransferase (**ALT**), Aspartate transaminase (**AST**), alkaline phosphatase (**ALP**), Phosphorus(P), Calcium (**Ca**), albumin (**ALB**), immunoglobulin A (**IgA**), IgM and IgG were detected with an automatic biochemical analyzer (ZY-450, KHB) using the corresponding kits. Serum levels of Interleukin-1 beta (**IL-1β**), Interleukin-6 (**IL-6**), Tumor necrosis factor-alpha (**TNF-α**), Interleukin-2 (**IL-2**), Follicle-stimulating hormone (**FSH**), luteinizing hormone (**LH**), Estradiol (**E2**), and Progesterone (**Prog**) were determined by the ELISA method according to the manufacturer's instructions (Nanjing Jiancheng Bioengineering Institute, Nanjing, China).

### Histology analysis

Ovarian, oviduct, duodenum, jejunum, and ileum fixed in 4% formaldehyde were used for morphological analysis via hematoxylin and eosin (**H&E**) staining. Following dehydration, embedding, sectioning, and staining, the tissue sections were examined under a light microscope. Villus height and crypt depth in the duodenum, jejunum, and ileum were measured using CaseViewer 2.4.0 software, and the villus height to crypt depth ratio (V/C) was calculated.

### Transcriptome sequencing

The total RNA from oviduct and cecum samples was extracted using an RNAiso Plus reagent (TaKaRa, Dalian, China) in accordance with the protocol of the manufacturer. Nanodrop 2000 was used to detect the concentration and purity of the extracted RNA, agarose gel electrophoresis was used to detect the integrity of RNA, and Agilent 2100 was used to determine the RNA Integrity Number value. RNA-seq libraries were prepared using the TruSeq Stranded mRNA LT Sample Prep Kit according to the specifications (Illumina, San Diego, USA) and then sequenced using an MGI high-throughput sequencer by the Frasergen Biotechnology Co., Ltd. (Wuhan, China). Raw sequencing reads were subjected to quality control to remove adaptor sequences, reads containing ambiguous bases (N > 0.5%), and low-quality reads (quality score < Q20), generating high-quality clean reads. Quality control procedures were applied according to standard RNA-seq data processing pipelines to ensure that only high-quality reads were retained for downstream analyses. Clean reads were aligned to the reference chicken genome using HISAT2 (v2.1.0) and mapped to reference transcript sequences using Bowtie2 (v2.3.5). Only reads passing quality control and successful alignment were used for expression quantification and subsequent differential expression analyses. Transcript-level read counts were quantified using RSEM (v1.3.1), followed by normalization to fragments per kilobase of transcript per million mapped reads (FPKM). Differentially expressed genes (**DEGs**) were identified using DESeq2 (v1.22.2), with an adjusted P value (false discovery rate, FDR) < 0.05 and absolute |log₂ FC| ≥ 1 considered statistically significant. GO enrichment analysis was conducted using GOseq (v1.22), and significantly enriched terms were defined by Q values < 0.05. KEGG pathway enrichment analysis was performed using KOBAS (v3.0), and pathways with Benjamini–Hochberg-corrected Q values < 0.05 were considered significantly enriched.

### Molecular docking

AutoDockTools was used to perform molecular docking verification on the main active ingredients and core target proteins of WEA. First, the protein structure of the core target was downloaded from the RCSB-PDB database, and the protein was converted to pdbqt format using OpenBabelGUI. The 2D structure of the active ingredients of WEA was downloaded from the PubChem database, saved in mol2 format, and converted to pdbqt format using OpenBabelGUI. The above targets and ingredients were visualized separately in AutoDockTools-1.5.6.

### Genes expression analysis

Total RNA was extracted from the ovary, oviduct, ileum, and cecum tissue samples using the Trizol reagent and quantified using an Ultramicro ultraviolet spectrophotometer (Thermo Fisher, NanoDrop One, Shanghai, China). The extracted RNA was then converted to cDNA via reverse transcription utilizing the RT Mix Kit with gDNA Clean for qPCR kits. RT-qPCR was performed using SYBR Green, and the relative mRNA expression of the target genes was calculated with β-actin as the internal control. The primers sequences are provided in Table s2.

### Determination of intestinal digesta SCFAs

The hindgut digesta of the selected laying hens was collected for determining short-chain fatty acids (**SCFAs**). The digesta (0.5g) was mixed with 5 mL of ultrapure water by vortexing for 30 min, refrigerated at 4°C overnight. After centrifugation at 10000 g for 10 min, 1350 μL of the supernatant was mixed with metaphosphoric acid solution (150 μL, 25%), stood at room temperature for 3h, and then centrifuged at 10000 g for 10 min. The supernatant was used to determine the SCFAs concentration in the digesta by using a gas chromatographic method.

### Sequencing of gut microbiota

Based on the OTU profiles, diversity indexes were calculated, and sequencing depth was evaluated. Community structure was statistically analyzed at different taxonomic levels using the obtained taxonomic information. Furthermore, additional statistical analyses and data visualization, including multivariate analysis and significance testing of differences in community composition and phylogenetic features, were conducted across multiple samples.

### Statistical analysis

SPSS23.0 statistical software was used to analyze the differences in production performance, egg quality, organ indexes, and serum biochemical parameters among different groups by Tukey-Kramer method, and the histological analysis data were analyzed by one-way ANOVA analysis. Based on the above data, WEA-L was used for independent sample T-test analysis in subsequent experiments and labeled as WEA. The results were expressed as "mean ± SEM", P<0.05 was deemed a significant difference, and 0.05<P<0.1 was considered as a trend toward significance. Data were graphed using GraphPad Prism 9.0 software.

## Results

### Chemical composition of WEA and network pharmacology

**Fig. 1:**
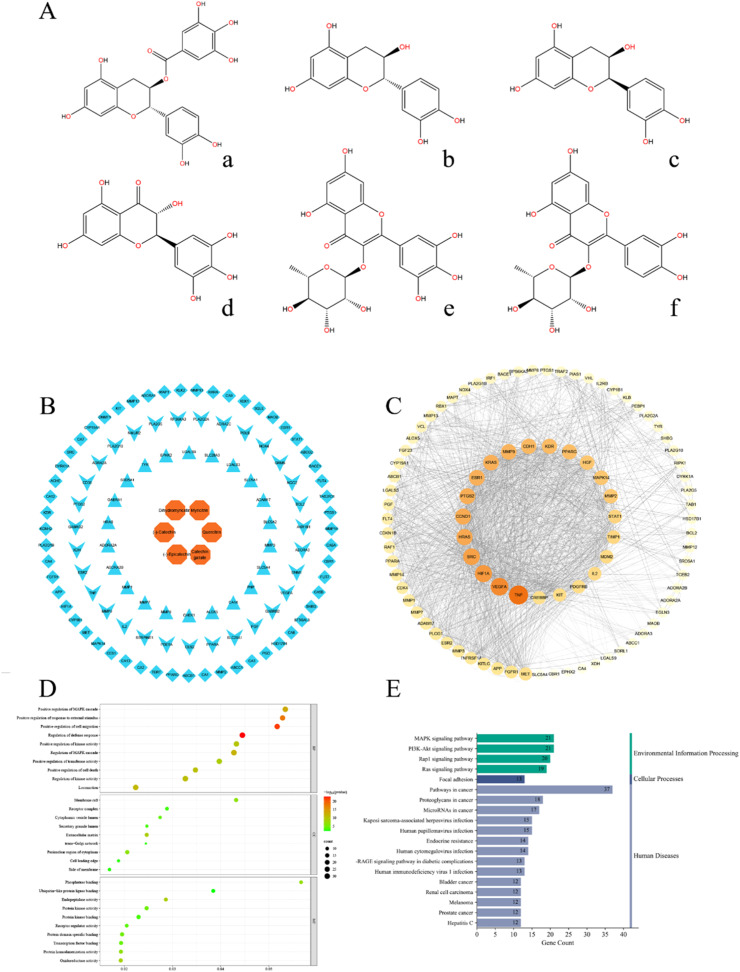
Network pharmacology. (A) WEA main compound composition structure ((a) (-)-Catechin gallate, (b) (-)-Catechin, (c) (-)-Epicatechin, (d) dihydromyricetin, (e) Myricitrin, (f) Quercitrin). (B) The component network of main compound components of WEA and the reproductive system aging targets. (C)The PPI network of main compound components of WEA and the reproductive system aging targets. (D) GO terms of hub genes. (E) Top 20 KEGG pathways of hub genes.

Using UPLC-MS/MS analysis, a total of 92 flavonoid compounds were identified in WEA. Based on their relative abundance, the top six flavonoids were determined to be dihydromyricetin, myricetin, (-)-catechin, quercetin, (-)-epicatechin, and (-)-catechin gallate (Fig. 1, Table s3). To explore how these predominant flavonoids may contribute to the mitigation of reproductive system aging, a "compound–target" interaction network was constructed linking the six major flavonoid constituents of WEA with 110 known targets associated with alleviating reproductive aging (Fig. 1B). These common targets were subsequently imported into the STRING database for network construction and analysis (Fig. 1C), which revealed key hub targets including tumor necrosis factor (**TNF**), vascular endothelial growth factor A (**VEGFA**), hypoxia inducible factor 1 subunit alpha (**HIF1A**), proto-oncogene tyrosine-protein kinase Src (**SRC**), and v-Ha-ras Harvey rat sarcoma viral oncogene homolog (**HRAS**). GO enrichment analysis of the 110 predicted targets yielded a total of 1,176 enriched GO terms, including 1,047 biological processes, 43 cellular components, and 86 molecular functions (Fig. 1D). KEGG pathway enrichment analysis further identified 149 signaling pathways associated with the potential therapeutic effects of WEA (Fig. 1E), among which the MAPK, PI3K/AKT, Rap1, and Ras signaling pathways were prominently represented. Notably, the core targets TNF, VEGFA, and HIF1A identified in the PPI network are closely associated with the PI3K/AKT signaling pathway. These findings suggest that WEA may exert its anti-reproductive-aging effects primarily through modulation of the PI3K/AKT pathway.

### Laying performance and egg quality

To evaluate the effects of WEA supplementation on the production performance of laying hens, different concentrations of WEA were added to a basal diet. The results indicated that there were no significant differences among the WEA-L, WEA-M, and WEA-H groups compared to the control group in terms of laying rate, average egg weight, malformed egg rate, average daily feed intake, or feed conversion ratio. However, there was a trend toward increased egg weight in the WEA groups, particularly compared to the control (P = 0.098, Table s4).Further analysis revealed that, relative to the control group, the WEA-L, WEA-M, and WEA-H groups exhibited significantly higher, egg shape index (P < 0.01), yolk color score (P < 0.05), and yolk index (P < 0.05), with haugh unit also showing an increasing trend (P = 0.096, Table s5). Notably, albumen height was significantly elevated in both the WEA-L and WEA-H groups (P < 0.05). In addition, compared to the control group, eggshell strength was significantly improved in the WEA-M group (P < 0.05), and eggshell thickness was significantly increased in both the WEA-L (P < 0.01) and WEA-M (P < 0.01) groups.

### WEA affects immune function and organ indexes

Compared with the control group, the WEA-M group showed a highly significant increase in serum IgG levels (P < 0.01), while the WEA-L group exhibited a significant elevation in ALB levels and a reduction in glucose (**GLU**) levels (P < 0.05). In addition, there was a tendency toward increased levels of IgA (P = 0.064) and Ca (P = 0.055) in all WEA-treated groups (Table 1).Moreover, the liver index was significantly elevated in all WEA-treated groups compared to the control (P < 0.01), and both the WEA-M and WEA-H groups showed a significant increase in spleen index (P < 0.05, Table 1). The oviduct index also exhibited an increasing trend in the WEA-L and WEA-H groups compared to the control (P = 0.076, Table s6).

**Table 1:** Effects of dietary WEA on serum biochemical parameters of laying hens.

	Mean±SEM				P-value
	Control	WEA-L	WEA-M	WEA-H	
IgM(g/L)	0.23±0.01	0.54±0.02	0.56±0.02	0.69±0.03	0.336
IgG(g/L)	0.01±0.00^b^	0.02±0.01^ab^	0.03±0.00^a^	0.02±0.00^ab^	0.006
IgA(g/L)	0.00±0.00	0.03±0.01	0.02±0.01	0.05±0.02	0.064
P(mmol/L)	3.02±0.17	3.53±0.24	3.29±0.23	3.18±0.24	0.438
Ca(mmol/L)	5.46±0.32	6.55±0.15	5.94±0.30	6.09±0.25	0.055
ALT(U/L)	6.86±1.57	5.43±1.86	5.71±1.53	7.22±2.53	0.889
AST(U/L)	282.36±15.50	274.23±25.62	290.24±15.88	304.98±18.15	0.708
ALP(U/L)	565.04±68.02	457.18±92.89	463.40±50.55	360.37±41.76	0.176
ALB(g/L)	25.33±1.33^b^	31.60±1.86^a^	29.00±1.57^ab^	28.24±0.94^ab^	0.043
GLU(mmol/L)	15.04±0.45^a^	12.45±0.56^b^	14.13±0.75^ab^	13.95±0.61^ab^	0.046

a,b Different lowercase letters in the same row indicate significant difference (p < 0.05), and the same letters or no letters indicate no significant difference (p > 0.05). Control: basal diet; WEA-L: basal diet+50mg/kg Water extract of *A. grossedentata*; WEA-M: basal diet+150mg/kg Water extract of *A. grossedentata*; WEA-H: basal diet+250mg/kg Water extract of *A. grossedentata*; SEM: standard error of the mean.

### WEA alters ovarian and fallopian tube morphology and the levels of sex hormones

**Fig. 2:**
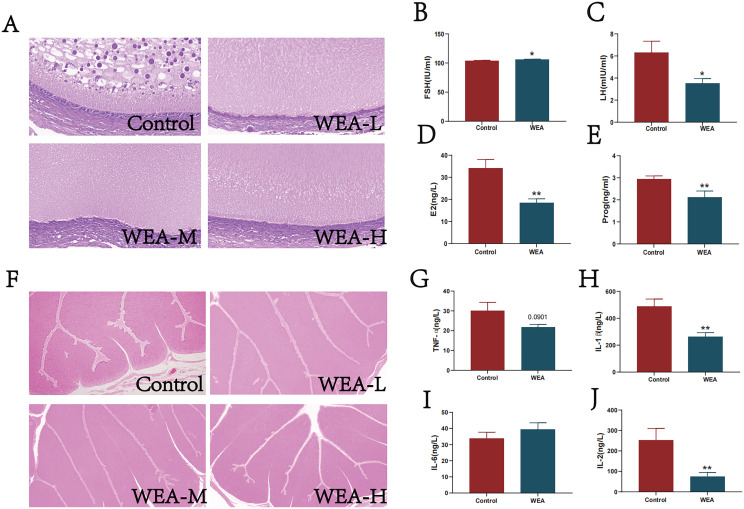
The histological representative images of the (A) ovary and (F) oviduct, all images are at 40x magnification. Serum sex hormone((B) FSH, (C) LH, (D) E2, and (E) Prog) and inflammatory factors((G)TNF-α, (H) IL-1β, (I) IL-6, and (J) IL-2) levels were measured by ELISA. Values were shown as mean±SEM, compared with Control *P<0.05,**P<0.01.

To further investigate the effects of WEA on reproductive organs, histological analyses of ovarian and oviduct were performed. Ovarian tissue sections from the control group revealed a reduced number of granulosa cells with loose and irregular cell arrangement. In contrast, dietary supplementation with WEA markedly improved these histological features, indicating enhanced ovarian integrity (Fig. 2A). Similarly, examination of oviduct histomorphology demonstrated that hens in the WEA-L and WEA-H groups exhibited long, thick folds in the ampullary region, while the WEA-M group showed tree-like, hypertrophic folds with a greater abundance of glandular structures. In comparison, the control group displayed sparse, loosely organized folds, suggestive of weaker secretory capacity (Fig. 2F). To further substantiate the protective role of WEA on ovarian function, serum sex hormone levels were assessed (Fig. 2B–E). Compared to the control group, WEA supplementation significantly increased serum levels of FSH (P < 0.05), while levels of LH (P < 0.05), E2 (P < 0.01), and Prog (P < 0.01) were significantly reduced. Additionally, WEA significantly suppressed pro-inflammatory cytokines IL-1β (P < 0.01) and IL-2 (P < 0.01), and showed a decreasing trend in TNF-α (P = 0.0901, Fig. 2G–J).

### WEA alters intestinal morphology

**Fig. 3:**
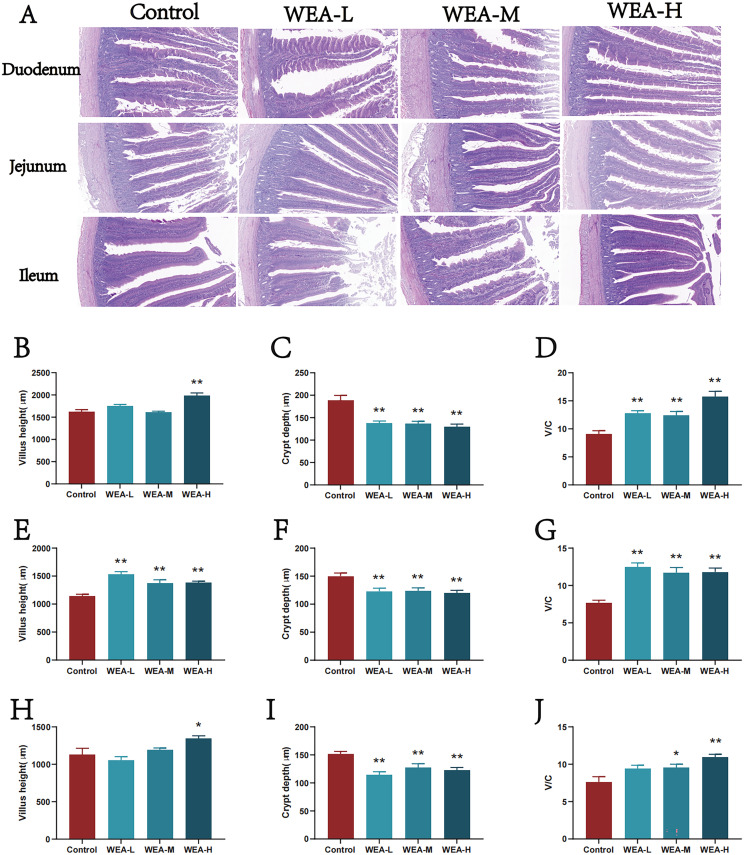
The histological representative images of the (A) duodenum, jejunum, and ileum, all images are at 5x magnification. (B) Duodenal villus height and (C) crypt depth and (D) ratio of villus height to crypt depth, (E) jejunal villus height and (F) crypt depth and (G) ratio of villus height to crypt depth, (H) ileal villus height and (I) crypt depth and (J) ratio of villus height to crypt depth. Values was shown as mean±SEM, Compared with Control *P<0.05,**P<0.01.

To assess the impact of WEA on intestinal health, intestinal morphology in laying hens was examined (Fig. 3A). The results showed that the duodenum, jejunum, and ileum of hens in the WEA-treated groups exhibited more intact structural integrity compared to the control group, with reduced villus shedding observed. Notably, WEA-H supplementation significantly increased villus height in the duodenum (P < 0.05, Fig. 3B). All WEA-treated groups (WEA-L, WEA-M, and WEA-H) exhibited reduced crypt depth (Fig. 3C) and significantly enhanced villus height-to-crypt depth ratio (P < 0.01) in the duodenum (Fig. 3D). In the jejunum, WEA treatment significantly restored villus height (P < 0.01, Fig. 3E) and villus height-to-crypt depth ratio (P < 0.01, Fig. 3G), while also reducing crypt depth in comparison to the control group (Fig. 3F). Similarly, in the ileum, WEA-H treatment significantly improved villus height (P < 0.05, Fig. 3H), and all WEA-treated groups significantly reduced crypt depth (P < 0.01, Fig. 3I). Moreover, both WEA-M (P < 0.05) and WEA-H (P < 0.01) groups demonstrated marked improvements in the villus height-to-crypt depth ratio (Fig. 3J).

### Analysis of differentially expressed genes at the transcriptomics level of laying hens and molecular docking

**Fig. 4:**
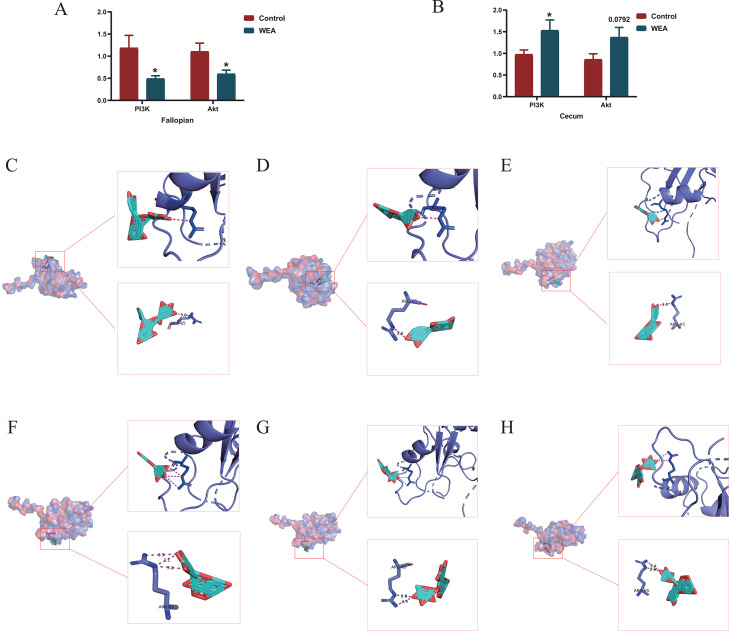
RT-PCR validation of WEA on PI3K/AKT expression in (A-B) oviduct, and cecum. Molecular docking: (C) (-)-Catechin gallate-PI3K; (D) (-)-Catechin-PI3K; (E) (-)-Epicatechin-PI3K; (F) (-)-Myricitrin-PI3K; (G) Dihydromyricetin-PI3K; (H) Quercitrin-PI3K.

To further elucidate the potential mechanisms by which WEA exerts its effects on reproductive and intestinal health, transcriptomic analyses were conducted across various tissues. Differential gene expression analysis between the control and WEA-treated groups revealed substantial changes in gene expression in the oviduct (5228 DEGs) and cecum (863 DEGs). Specifically, in the oviduct, 2,563 genes were upregulated and 2,665 downregulated (Figure s1A) and in the cecum, 494 genes were upregulated and 369 downregulated (Figure s1B). Subsequent KEGG pathway enrichment analysis of these DEGs was performed, and the top 20 most significantly enriched pathways were visualized using bubble plots (Figure s1C–F). Notably, both upregulated and downregulated DEGs in the oviduct and cecum were enriched in the PI3K/AKT signaling pathway when compared with the control group. Based on these transcriptomic findings, in combination with prior network pharmacology analysis, the PI3K/AKT signaling pathway was selected for further investigation as a key regulatory mechanism underlying the beneficial effects of WEA. Two key genes within the PI3K/AKT signaling pathway—PI3K and AKT—were selected for validation via RT-qPCR. The results confirmed that the expression patterns of these genes were consistent with the RNA-seq data (Fig. 4A–B), supporting the reliability of the transcriptomic findings. Furthermore, molecular docking analysis was performed to assess the binding affinity between the major flavonoid components of WEA and the PI3K protein. The docking scores for the interactions ranged from –7.129 to –6.796 kJ/mol (Table s7), indicating favorable binding energies. Visualization of the molecular docking models further illustrated the interaction patterns between the flavonoids and PI3K (Fig. 4C-H).These results collectively suggest that the flavonoid constituents of WEA may directly interact with PI3K, thereby potentially contributing to the modulation of the PI3K/AKT pathway.

### WEA alleviates ovarian apoptosis and enhances the anti-inflammatory function of oviduct

**Fig. 5:**
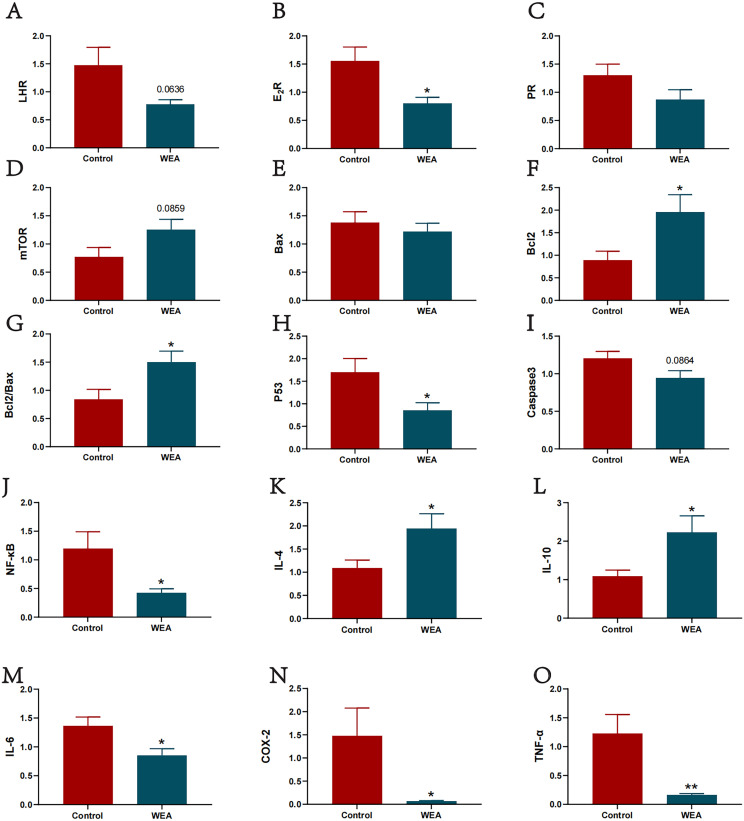
(A) LHR, (B) E2R, (C) PR, (D) mTOR, (E) Bax, (F) Bcl2, (G) Bcl2/Bax, (H) P53 and (I) Caspase3 expression in the ovary of laying hens treated with WEA. (J) NF-κB, (K) IL-4, (L) IL-10, (M) IL-6, (N) COX-2, (O) TNF-α expression in the oviduct of laying hens treated with WEA. Values were shown as mean±SEM, Compared with Control *P<0.05,**P<0.01.

To further investigate the mechanism of action of WEA on reproductive function, this study explored the effect of WEA on ovarian mTOR signaling. Compared with the control group, the WEA group showed significantly downregulated expression of E2R (P < 0.05, Fig. 5B) and P53 (P < 0.05, Fig. 5H), while significantly upregulated expression of Bcl2 (P < 0.05, Fig. 5F) and Bcl2/Bax (P < 0.05, Fig. 5G). Furthermore, the expression of LHR (P = 0.0636, Fig. 5A) and Caspase3 (P = 0.0864, Fig. 5I) in the WEA group tended to be lower than that in the control group, while the expression of mTOR (P = 0.0859, Fig. 5D) in the WEA group tended to be higher than that in the control group. This suggests that WEA may regulate sex hormone levels by modulating ovarian sex hormone receptor gene expression and effectively activating the mTOR pathway, thereby regulating the expression of downstream apoptotic genes, exerting an anti-apoptotic effect to a certain extent, delaying ovarian aging. Then, the effect of WEA on NF-κB in the oviduct was investigated. Compared to the control group, the expression of pro-inflammatory mediators NF-κB (P < 0.05, Fig. 5J), IL-6 (P < 0.05, Fig. 5M), Cyclooxygenase-2 (**COX-2**) (P < 0.05, Fig. 5N), and TNF-α (P < 0.01, Fig. 5O) was significantly reduced in the WEA group. Conversely, the anti-inflammatory cytokines interleukin-4 (**IL-4**; P < 0.05, Fig. 5K) and interleukin-10 (**IL-10**; P < 0.05, Fig. 5L) were significantly upregulated.

### WEA improves intestinal function

**Fig. 6:**
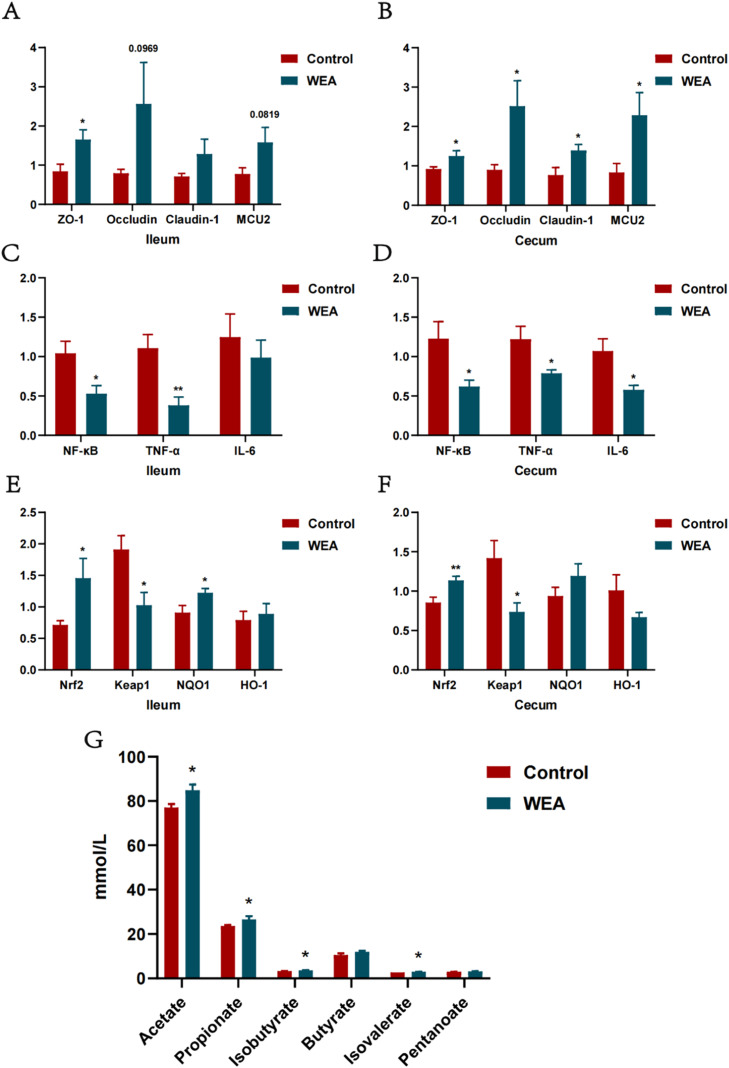
(A-B) ZO-1, Occludin, Claudin-1 and MUC2; (C-D) NF-κB, TNF-α and IL-6; (E-F) Nrf2, Keap1, NQO1 and HO-1 expression in the ileum and cecum of laying hens treated with WEA; (G) Content of short-chain fatty acids in the hindgut of laying hens. Values were shown as mean±SEM, compared with Control *P<0.05,**P<0.01.

In this study, the expression of genes associated with intestinal barrier integrity was evaluated in the ileum and cecum of laying hens. Compared to the control group, laying hens supplemented with WEA exhibited significantly upregulated expression of Zonula Occludens-1 (**ZO-1**) in the ileum (P < 0.05, Fig. 6A). Additionally, Occludin (P = 0.0969) and mitochondrial calcium uniporter 2 (**MUC2**) (P = 0.0819) also showed an upward trend in expression. In the cecum, the expression of tight junction-related genes including ZO-1, Occludin, Claudin-1, and MUC2 was significantly elevated in the WEA group (P < 0.05, Fig. 6B), indicating that WEA supplementation contributes to enhanced intestinal barrier function. To further investigate the anti-inflammatory potential of WEA, expression of NF-κB-associated inflammatory genes was assessed. In the ileum, the expression of NF-κB (P < 0.05) and TNF-α (P < 0.01) was significantly downregulated in the WEA group (Fig. 6C). Similarly, in the cecum, WEA significantly reduced the expression levels of NF-κB, TNF-α, and IL-6 (all P < 0.05, Fig. 6D). Given the interplay between oxidative stress and inflammation, this study also evaluated the expression of Nrf2-related antioxidant genes in the ileum and cecum. In the ileum, WEA supplementation significantly upregulated nuclear factor erythroid 2-related factor 2 (**Nrf2**) and NAD(P)H quinone dehydrogenase 1 (**NQO1**) (both P < 0.05), while significantly downregulating Keap1 (P < 0.05, Fig. 6E). A similar expression pattern was observed in the cecum, where Nrf2 was significantly upregulated (P < 0.01) and Keap1 was downregulated (P < 0.05, Fig. 6F). In addition, SCFAs profiles in the hindgut were analyzed to assess microbial metabolic activity. The concentrations of acetic acid, propionic acid, isobutyric acid, and isovaleric acid were significantly higher in the WEA group compared to the control (P < 0.05), whereas levels of butyric acid and valeric acid showed no significant differences (Fig. 6G).

### WEA alters the gut microbiota

To investigate the impact of WEA supplementation on the gut microbiota of laying hens, 16S rDNA amplicon sequencing was conducted. Compared to the control group, there were no significant differences in the Shannon and Simpson indices in the foregut of the WEA group (Figure s2A–B). However, in the hindgut, the Shannon index was significantly increased in the WEA group (P < 0.05, Figure s2D), and the Simpson index showed an increasing trend (P = 0.08, Figure s2E). Principal coordinate analysis revealed no significant differences between groups in the foregut (Figure s2G), whereas a significant separation was observed in the hindgut (P < 0.05, Figure s2I), which was consistent with the findings from non-metric multidimensional scaling analysis (Figure s2H, s2J).

**Fig. 7:**
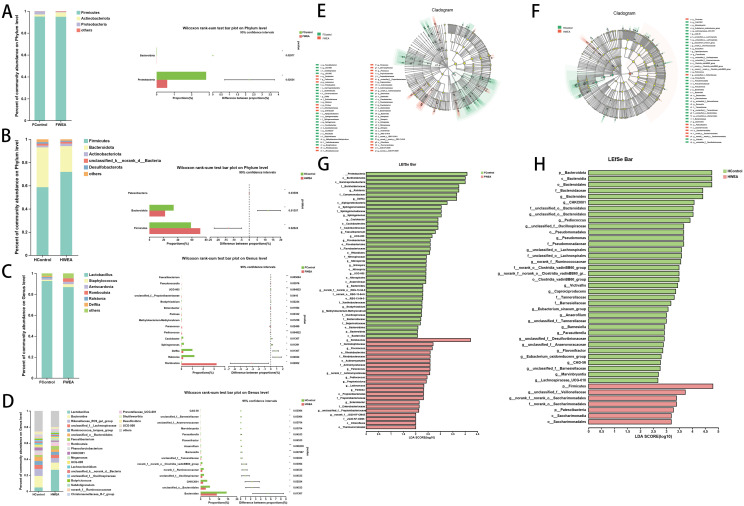
Effects of WEA on the composition of gut microbiota of laying hens. (A) Relative abundance of foregut in gut microbiota at the phylum level; (B) Relative abundance of hindgut in gut microbiota at the phylum level; (C) Relative abundance of foregut in gut microbiota at the genus level; (D) Relative abundance of hindgut in gut microbiota at the genus level; (E) The specific gut microbial taxa from phylum to genus in the foregut; (F) The specific gut microbial taxa from phylum to genus in the hindgut; (G) The LDA scores are computed for features at the OTU level in the foregut; (H) The LDA scores are computed for features at the OTU level in the hindgut. (LDA> 2). *P<0.05,**P<0.01.

At the phylum level, analysis of the foregut microbiota (Fig. 7A) revealed that the dominant bacterial phyla were Firmicutes, Actinobacteriota, and Proteobacteria, collectively accounting for over 90% of the total microbial abundance. Comparison of relative abundance between the control and WEA groups showed that the abundance of Bacteroidota and Proteobacteria was reduced in the WEA group (Fig. 7A). In the hindgut (Fig. 7B), the dominant phyla included Firmicutes, Bacteroidota, Actinobacteriota, unclassified_k_norank_d_Bacteria, and Desulfobacterota. Relative abundance analysis (Fig. 7B) indicated that the WEA group exhibited an increased abundance of Patescibacteria and Firmicutes, while Bacteroidota was decreased compared to the control group.

At the genus level, Lactobacillus was identified as the predominant genus in the foregut, comprising over 80% of the total microbial community (Fig. 7C). Compared to the control group, the WEA group exhibited significantly decreased relative abundances of Faecalibacterium, UCG-005, Bradyrhizobium, Methylobacterium-Methylorubrum, Caulobacter, Sphingomonas, Delftia, and Ralstonia (P < 0.05), while the abundances of Pseudonocardis, unclassified_f_Propionibacteriaceae, Enterobacter, Pantoea, Paracoccus, Pediococcus, and Romboutsia were significantly increased (P < 0.05) (Fig. 7C). In the hindgut, the dominant genera were Lactobacillus, Bacteroides, and Rikenellaceae_RC9_gut_group, accounting for more than 50% of the total microbial abundance (Fig. 7D). Relative abundance analysis between groups (Fig. 7D) showed that the WEA group had significantly lower levels (P < 0.05) of CAG-56, unclassified_f_Barnesiellaceae, unclassified_f_Anaerovoracaceae, Marvinbryantia, Parasutterella, Flavonifractor, Anaerofilum, Barnesiella, unclassified_f_Tannerellaceae, norank_f_norank_o_Clostridia_vadinBB60_group, norank_f_Ruminococcaceae, unclassified_f_Oscillospiraceae, CHKCIO01, unclassified_o_Bacteroidales, and Bacteroides. Additionally, Lactobacillus exhibited an increasing trend in the WEA group (P = 0.0761).

LEfSe analysis further revealed that the foregut of the WEA group was enriched in Romboutsia, f_Dermatophilaceae, Piscicoccus, o_Rhodobacterales, f_Rhodobacteraceae, o_Actinomycetales, f_Actinomycetaceae, Paracoccus, g_norank_f_Actinomycetaceae, Pediococcus, Propioniciclava, Lactococcus, Pantoea, o_Propionibacteriales, f_Propionibacteriaceae, Enterobacter, f_Enterobacteriaceae, g_unclassified_f_Propionibacteriaceae, g_norank_f_JG30-KF-CM45, f_JG30-KF-CM45, c_Chloroflexia, and o_Thermomicrobiales (Fig. 7G). In contrast, the hindgut of the WEA group was enriched in p_Firmicutes, g_unclassified_f_Veillonellaceae, g_norank_f_norank_o_Saccharimonadales, f_norank_o_Saccharimonadales, p_Patescibacteria, c_Saccharimonadia, and o_Saccharimonadales (Fig. 7H).

### Correlation analysis

**Fig. 8:**
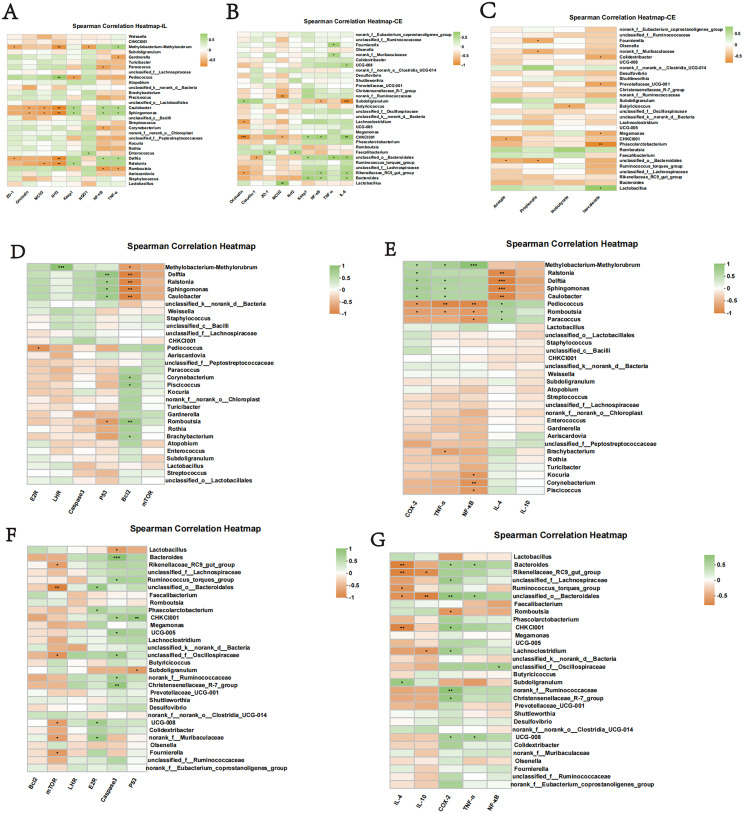
Key genera involved in mediating sex hormone receptor, apoptosis, pro-inflammatory, intestinal barrier, antioxidant, and short-chain fatty acids genes in the ovary, oviduct, ileum, and cecum. Association analysis of (A) ileum and foregut microbiota; (B) association analysis of cecum, (C) hindgut short-chain fatty acids and hindgut microbiota; (D) association analysis of ovary, (E) oviduct and foregut microbiota; association analysis of (F) ovary, (G) oviduct and hindgut microbiota. Green color represents significant positive correlation and orange color represents significant negative correlation, and the independent right color bars depict correlation coefficients between the microbiota and genes. The correlation was considered significant when the absolute value of Spearman's rank correlation coefficient was >0.6 and statistically significant (P< 0.05). The significance is indicated: *P< 0.05, **P< 0.01, ***P< 0.001.

Based on the results of rank-sum tests and LEfSe analysis, several representative bacterial taxa in the foregut and hindgut of the WEA group were identified for further correlation analysis, including Methylobacterium-Methylorubrum, Delftia, Ralstonia, Bacteroides, CHKCI001, and unclassified_f_Oscillospiraceae. Correlation analysis between foregut microbiota and ileal gene expression (Fig. 8A) revealed that Methylobacterium-Methylorubrum was negatively correlated with ZO-1, Nrf2, and NQO1, but positively correlated with TNF-α. Pediococcus was positively correlated with Nrf2 and negatively correlated with Keap1. Additionally, Caulobacter and Sphingomonas exhibited negative correlations with Occludin, MUC2, and Nrf2, and positive correlations with Keap1, NF-κB, and TNF-α. Delftia was negatively associated with ZO-1 and Nrf2, and positively with NF-κB and TNF-α. Ralstonia was negatively correlated with MUC2 and Nrf2, and positively correlated with Keap1. Regarding hindgut microbiota and ileal gene expression (Fig. 8B), norank_f_Ruminococcaceae showed a negative correlation with MUC2, while CHKCI001 was negatively correlated with Occludin and MUC2, but positively correlated with Keap1, NF-κB, and IL-6. Unclassified_o_Bacteroidales was negatively correlated with Claudin-1 and positively with Keap1, TNF-α, and IL-6. Bacteroides was positively correlated with Keap1, NF-κB, and IL-6. Correlation analysis between hindgut microbiota and SCFAs in hindgut contents (Fig. 8C) showed that Lactobacillus was positively correlated with isovalerate. Correlation analysis between foregut bacteria and ovary genes showed (Fig. 8D) that Methylobacterium-Methylorubrum was positively correlated with LHR and negatively correlated with P53. Delftia, Ralstonia, Sphingomonas, and Caulobacter were positively correlated with P53 and negatively correlated with Bcl2. Pediococcus was negatively correlated with E2R. Romboutsia was positively correlated with Bcl2 and negatively correlated with P53. Piscicoccus was positively correlated with Bcl2. In terms of correlation between foregut microbiota and oviduct gene expression (Fig. 8E), Methylobacterium-Methylorubrum was positively correlated with NF-κB, TNF-α, and COX-2. Ralstonia showed a positive correlation with COX-2 and a negative correlation with IL-4. Delftia, Sphingomonas, and Caulobacter were negatively associated with IL-4, and positively with TNF-α and COX-2. Conversely, Pediococcus and Romboutsia were negatively correlated with NF-κB, TNF-α, and COX-2, while positively correlated with IL-4. Paracoccus was negatively associated with NF-κB and positively with IL-4, and Piscicoccus also exhibited a negative correlation with NF-κB. Correlation analysis of hindgut bacteria and ovary genes showed (Fig. 8F) that Bacteroides and norank_f_Ruminococcaceae were positively correlated with Caspase3. CHKCI001 was positively correlated with Caspase3 and P53. Unclassified_o_Bacteroidales was positively correlated with E2R and negatively correlated with mTOR. Unclassified_f_Oscillospiraceae was positively correlated with Caspase3 and negatively correlated with mTOR. Finally, in the correlation analysis between hindgut microbiota and oviduct expression (Fig. 8G), Bacteroides showed positive correlations with COX-2 and TNF-α, and a negative correlation with IL-4. Unclassified_o_Bacteroidales was positively correlated with COX-2 and TNF-α, and negatively correlated with IL-4 and IL-10. CHKCI001 exhibited a positive correlation with COX-2 and a negative correlation with IL-4. Unclassified_f_Oscillospiraceae was positively correlated with NF-κB, while norank_f_Ruminococcaceae showed a positive correlation with COX-2.

## Discussion

In this study, WEA was systematically characterized and combined with network pharmacology to explore its potential involvement in reproductive system aging. The detection of 92 flavonoid-related metabolites, with dihydromyricetin, myricetin, (-)-catechin, quercetin, (-)-epicatechin, and (-)-catechin gallate as the predominant components, indicates that the biological activity of WEA is likely attributable to the coordinated actions of multiple constituents rather than a single dominant compound. The network pharmacology results demonstrated a substantial intersection between the predicted targets of WEA flavonoids and genes previously implicated in reproductive aging. Within the PPI network, TNF, VEGFA, and HIF1A emerged as highly connected nodes, implying that inflammatory signaling, vascular regulation, and cellular stress responses may represent key biological processes influenced by WEA (Khajouei et al., 2021). These processes are well recognized as contributors to functional decline in reproductive tissues during aging. KEGG pathway enrichment analysis indicated that the MAPK and PI3K/AKT signaling pathways were significantly enriched. Given that the PI3K/AKT pathway is associated with the core targets TNF, VEGFA, and HIF1A, it is postulated that this pathway may play a central role in the anti-aging effects of WEA on the reproductive system. Pathway enrichment analysis further revealed significant involvement of the PI3K/AKT signaling cascade, which integrates several of the identified hub targets into a unified regulatory framework.

In this study, WEA was incorporated into the diets of laying hens to evaluate its potential effects on reproductive health and production performance. The results indicated that dietary supplementation with WEA had no significant impact on general production performance; however, notable improvements in egg quality parameters were observed. Specifically, enhancements were recorded in egg weight, shape index, yolk color, albumen height, shell strength, shell thickness, and yolk index. Additionally, serum calcium levels in the WEA group exhibited an increasing trend compared to the control group. Given the critical role of calcium in determining eggshell quality, this change may be associated with, at least in part, the observed improvements in shell-related traits (Xiao et al., 2019). Furthermore, hens receiving WEA demonstrated significantly elevated liver and spleen indexes, alongside a trend toward increased oviduct index, which were accompanied by changes in organ indexes related to reproductive tissues. Flavonoids have been widely recognized for their immunomodulatory properties in both cellular and animal models, including the activation of immune cells and promotion of T cell generation (Niu et al., 2018; Mitani et al., 2019). Among immunoglobulins, IgA, IgG, and IgM are considered the principal subtypes involved in immune regulation in poultry (Moon et al., 2021). In this context, the present study measured serum levels of these immunoglobulins and found that WEA supplementation significantly increased IgG concentrations and showed a trend toward elevated IgA levels. These findings indicate that dietary WEA was associated with altered immune-related indicators in laying hens, which is consistent with the observed increase in spleen index and suggests a potential immunomodulatory effect of WEA. To further assess hepatic safety, serum biochemical parameters related to liver function (ALT, AST, ALB, and ALP) were evaluated. No significant differences were detected among treatment groups, indicating that WEA supplementation was not associated with detectable hepatotoxic effects under the experimental conditions. Collectively, these findings suggest that dietary WEA was accompanied by improvements in egg quality and immune-related parameters, which may be relevant to reproductive system health. FSH plays a central role in regulating follicular development and maturation in laying hens, while LH promotes progesterone secretion, and E2 regulates folliculogenesis through feedback on the hypothalamic–pituitary axis (Darabi et al., 2020). As such, serum levels of FSH, LH, and E2 are considered sensitive biomarkers of reproductive performance in aging hens. In this study, WEA supplementation led to increased FSH and decreased LH, E2, and progesterone levels, which were observed in parallel with changes in reproductive-related indicators. Histological analyses further supported these observations. Ovarian tissues of hens fed WEA exhibited more orderly granulosa cell arrangements and improved ovarian morphology. Similar improvements were observed in the oviduct.

Moreover, WEA supplementation significantly reduced the serum levels of pro-inflammatory cytokines TNF-α, IL-1β, and IL-2, indicating a lower systemic inflammatory status associated with WEA intake. It should be noted that these phenotypic, hormonal, and inflammatory changes were identified based on associative evidence and do not, by themselves, establish causal relationships. Further mechanistic validation will be required to clarify their direct biological interconnections. The intestinal mucosa serves as a critical barrier against harmful substances and pathogenic microorganisms, while also playing an essential role in nutrient digestion and absorption. An increased villus height to crypt depth ratio, as well as taller villi and shallower crypts, is generally associated with improved nutrient absorption efficiency (Erben et al., 2014). In the present study, dietary supplementation with WEA led to an increase in villus height and the villus height-to-crypt depth ratio, alongside a reduction in crypt depth in the duodenum, jejunum, and ileum, compared to the control group. These histological alterations are consistent with improved intestinal morphological features that are commonly associated with enhanced nutrient absorption efficiency. This is consistent with findings from other flavonoid compounds, such as puerarin flavonoids, Allium mongolicum flavonoids, citrus flavonoids, and catechins, all of which have demonstrated beneficial effects on intestinal morphology (Chen, 2020; Erben et al., 2014; Liu, 2022; Xue et al., 2021). To further explore the underlying molecular mechanisms, transcriptomic sequencing was performed on the oviduct and cecum. A substantial divergence in transcriptomic profiles was observed between the WEA and control groups. Differential gene expression analysis revealed that WEA supplementation led to the upregulation of 2,563 DEGs in the oviduct and 494 DEGs in the cecum, and the downregulation of 2,665 DEGs in the oviduct and 369 DEGs in the cecum, respectively. Functional enrichment analyses further indicated that many of these DEGs were significantly enriched in the PI3K/AKT signaling pathway across all tissues. The PI3K/AKT pathway is a pivotal signaling cascade involved in the regulation of diverse cellular processes, including angiogenesis, metabolism, proliferation, survival, protein synthesis, transcription, and apoptosis. PI3K, the upstream initiator kinase, can be activated by various stimuli, such as tyrosine kinase receptors and G protein-coupled receptors. Upon activation, PI3K catalyzes the conversion of phosphatidylinositol to phosphatidylinositol-3,4,5-triphosphate [PI(3,4,5)P3], which functions as a secondary messenger to recruit and activate downstream effectors, notably AKT (also known as protein kinase B). Activated AKT mediates a range of cellular processes, making the PI3K/AKT pathway a critical regulator of both reproductive and intestinal health (Thorpe et al., 2015). Previous studies support the modulatory role of flavonoids in this pathway. For instance, myricitrin has been shown to inhibit LOX-1 expression and enhance activation of the STAT3 and PI3K/AKT/eNOS pathways, thereby attenuating ox-LDL-induced endothelial apoptosis (Qin et al., 2015). Orally administered myricitrin also exerts anti-inflammatory effects in DSS-induced acute colitis in mice by suppressing PI3K/AKT phosphorylation (Schwanke et al., 2013). Similarly, quercetin mitigates LPS-induced depressive-like behaviors in mice, possibly through inhibition of the PI3K/AKT/NF-κB inflammatory axis and associated alterations in neuroplasticity (Sun et al., 2021). Taken together, these observations suggest that the beneficial effects of WEA observed in the present study are associated with transcriptomic changes involving the PI3K/AKT signaling pathway, although causality cannot be inferred from the current data alone.

To further validate the results obtained in this study, qPCR was employed to investigate the effects of WEA on the PI3K/AKT signaling pathway, with a particular focus on anti-apoptotic, anti-inflammatory, and antioxidant mechanisms. Previous studies have demonstrated that AKT, once activated by PI3K, can phosphorylate multiple downstream targets, including mTOR, NF-κB, and Nrf2, thereby regulating cellular metabolism, growth, and survival. Based on these findings, the present study extended its investigation to examine whether WEA was associated with transcriptional changes in downstream components related to the PI3K/AKT axis. qPCR results showed that WEA significantly reduced the expression of E2R and LHR in the ovaries, which is consistent with the observed changes in serum sex hormone levels. Furthermore, the mTOR pathway plays a crucial role in follicular development. In the present study, alterations in the expression of mTOR-related genes suggest that WEA supplementation was associated with changes in ovarian apoptosis-related regulatory pathways. Moreover, WEA significantly reduced the expression of NF-κB and the expression of IL-6, COX-2, and TNF-α in the oviduct, while increasing the expression of IL-4 and IL-10. These expression patterns are indicative of a shift toward an anti-inflammatory transcriptional profile associated with WEA supplementation. During the laying period, hens are susceptible to reproductive tract inflammation. Flavonoids, known for their anti-inflammatory properties, can inhibit the activity of pro-inflammatory enzymes. Previous research demonstrated that epicatechin alleviates MSU-induced acute gouty arthritis both in vitro and in vivo by inhibiting the NLRP3 inflammasome and the NF-κB signaling pathway (Wu et al., 2022b). In light of these findings, the present results suggest that WEA may be associated with modulation of mTOR- and NF-κB-related signaling pathways in reproductive tissues; however, these observations are based on transcriptional evidence and do not establish direct causal mechanisms.

The gastrointestinal tract functions as a critical innate barrier that maintains internal homeostasis and prevents the invasion of pathogens and toxins. A structurally intact epithelial barrier is essential for preserving intestinal equilibrium and tolerance. Several in vitro and in vivo studies have shown that flavonoids can upregulate the expression of tight junction proteins, thus supporting the physical integrity of the intestinal epithelium (Mayangsari et al., 2021; Amevor et al., 2022). In the current study, WEA upregulated tight junction protein expression in the ileum and cecum. Previous research also indicated that quercetin enhanced intestinal barrier function in Caco-2 cells by increasing the expression of ZO-1 and occludin (Wan et al., 2021). Given that disruption of the intestinal barrier can trigger gut inflammation, WEA supplementation was associated with decreased expression of pro-inflammatory cytokines, potentially via modulation of the NF-κB signaling pathway. These results align with previous studies from our group, which demonstrated the anti-inflammatory effects of WEA in murine models of colitis. In addition, oxidative stress and inflammation are interrelated processes. To explore potential antioxidative effects, expression of key antioxidant genes (Nrf2, Keap1, and NQO1) was examined, showing higher expression in WEA-treated hens relative to controls, suggesting activation of the Nrf2/Keap1 pathway. This is consistent with prior research supporting the antioxidative properties of flavonoids via modulation of the Nrf2 pathway (Xie et al., 2019). Therefore, these findings suggest that WEA supplementation was associated with improved intestinal health, potentially through regulation of the Nrf2 and NF-κB pathways.

SCFAs, including acetate, propionate, and butyrate, are critical metabolic products for maintaining intestinal homeostasis, preserving epithelial barrier integrity, regulating intestinal gene expression, and modulating the gut microbial ecosystem (Parada Venegas et al., 2019; Yao et al., 2022). Flavonoids have been reported to exert regulatory effects on SCFA metabolism similar to those of beneficial gut microbiota (Zhao et al., 2021). This study employed gas chromatography to quantify SCFA concentrations in hindgut digesta. Dietary WEA supplementation was associated with elevated SCFA levels in the hindgut, consistent with prior observations of flavonoid-mediated microbiota-derived metabolite changes (Chen et al., 2021). Numerous studies have shown that intestinal microbiota is closely related to gut health and nutrient absorption (Liu et al., 2021).

16S rRNA sequencing was used to assess the potential effects of WEA on microbial communities in the foregut and hindgut. Observed changes suggest a limited but potentially relevant regulatory influence of WEA on gut microbiota composition. Dietary WEA did not significantly alter α-diversity in the foregut but was associated with changes in hindgut α-diversity. β-diversity analyses showed separation in microbial community structures between WEA and control groups in both foregut and hindgut. At the phylum level, Bacteroidetes and Firmicutes were dominant, consistent with previous studies (Khan et al., 2020). The gut microbiota, comprising commensals, probiotics, and potential pathogens, plays a crucial role in host health (Li et al., 2021). At the genus level, the foregut of the WEA group exhibited a higher relative abundance of beneficial bacteria, including Lactococcus, Enterobacter, Pantoea, and Paracoccus. These beneficial microbes may promote nutrient absorption through direct or indirect mechanisms. For example, Lactobacillus species are recognized for their capacity to reduce intestinal toxins, inhibit pathogenic growth, enhance gut barrier function, and mitigate inflammatory responses (Ge et al., 2020). Similarly, Enterobacter has been implicated in maintaining mucosal immune homeostasis, while Pantoea, an anaerobic genus, has shown potential benefits in improving quality of life. Moreover, biomarkers related to immune and antioxidant capacity positively correlated with the relative abundance of Paracoccus (Wang et al., 2022).

Conversely, several opportunistic and pathogenic bacteria were identified in the foregut microbiota, with reduced relative abundances in the WEA group, including Faecalibacterium and Methylobacterium-Methylorubrum.. In this study, correlation analyses indicate that Methylobacterium-Methylorubrum abundance was negatively associated with gut barrier markers (ZO-1, Nrf2, NQO1) and positively with TNF-α, suggesting a potential link to oxidative stress and inflammation. Furthermore, reproductive function has been associated with alterations or dysbiosis in gut microbial diversity and composition (Zhou et al., 2020). This study further analyzed the key genera associated with molecular markers in the ovary and oviduct. The results showed that Methylobacterium-Methylorubrum was positively correlated with LHR in the ovary, negatively correlated with Bcl2, and positively correlated with NF-κB, TNF-α, and COX-2 in the oviduct. Therefore, this study proposes that WEA may alleviate oxidative stress and inflammatory responses in the reproductive tract in conjunction with changes in the relative abundance of Methylobacterium-Methylorubrum in the foregut. Similarly, various pathogenic and opportunistic bacteria were detected in the hindgut microbiota, including Marvinbryantia, Parasutterella, and Flavonifractor, whose relative abundances were decreased in the WEA group. These genera are often observed in patients and have been implicated in detrimental health effects. Previous research has linked the pro-inflammatory genus Marvinbryantia to intestinal inflammation and gut dysfunction (Wang et al., 2020). Likewise, Parasutterella has been associated with the pathogenesis of chronic intestinal inflammation, while Flavonifractor shows a negative correlation with antioxidant enzymes, suggesting a potential role in promoting oxidative processes (Chen et al., 2018; Zhang et al., 2021). Notably, WEA supplementation downregulated the abundance of Bacteroides in the hindgut. The role of Bacteroides is complex and dualistic; although a common symbiont, it may act as a conditional pathogen, causing invasive infections. Certain Bacteroides species have been positively correlated with intestinal inflammation. Consistent with this, the present study found Bacteroides was positively correlated with intestinal Keap1, NF-κB, and IL-6 expression. In addition, Bacteroides was positively correlated with Caspase3 in the ovary. Bacteroides was positively correlated with TNF-α and COX-2, and negatively correlated with IL-4 in the oviduct, and correlation analyses indicated associations with intestinal antioxidant and inflammatory markers, as well as ovarian and oviduct gene expression. Furthermore, other Bacteroides species are involved in numerous metabolic processes and maintaining intestinal microenvironmental homeostasis, such as carbohydrate digestion and short-chain fatty acid production. For instance, commensal species including *Bacteroides acidifaciens, Bacteroides ovatus*, and *Bacteroides eggerthii* have demonstrated prebiotic regulatory functions beneficial to the host gut (Bie et al., 2021; Huang et al., 2021). Thus, the specific effects of Bacteroides require further detailed investigation.

Although this study integrates phenotypic, transcriptomic, and microbiome analyses to investigate the effects of WEA supplementation, several limitations should be acknowledged. In particular, the associations observed between specific bacterial taxa and host gene expression were derived from correlation analyses and therefore do not establish causal relationships. While these findings suggest potential microbiota–host interactions related to PI3K/AKT signaling, oxidative stress, and inflammatory regulation, additional functional validation is required to confirm direct mechanistic links. Future studies employing targeted microbial manipulation strategies, such as antibiotic-mediated microbiota depletion, fecal microbiota transplantation, or pathway-specific inhibition, will be necessary to elucidate whether the identified bacterial genera directly contribute to the regulation of host signaling pathways and reproductive aging. Also, a limitation of this study is the relatively small number of biological replicates (n = 8 per group) used for transcriptomic, microbiome, histological, and biochemical analyses, which may limit statistical power. This sampling strategy was based on the use of one hen per replicate to maintain independence of biological samples and avoid pseudo-replication, as well as practical considerations related to animal ethics and the cost of high-throughput analyses. Although these factors constrained the sample size, multiple lines of evidence, including production performance, reproductive traits, biochemical indicators, intestinal morphology, microbiome profiles, and transcriptomic pathways, consistently supported the effects of WEA supplementation. Nevertheless, future studies with larger sample sizes are warranted to further validate and extend these findings.

Taken together, the findings suggest that WEA supplementation is accompanied by coordinated changes in intestinal microbiota composition, host gene expression, and reproductive-related phenotypes in laying hens, particularly through reducing the relative abundance of foregut Methylobacterium-Methylorubrum and hindgut Bacteroides. This microbial modulation may influence the expression of the PI3K/AKT signaling pathway and its downstream effectors, which were correlated with reduced oxidative stress and inflammatory markers; however, causal relationships require further validation.

## Conclusion

In laying hen production, factors such as aging, nutritional imbalance, and sustained high productivity can induce damage to the reproductive system, leading to decreased reproductive capacity and consequent declines in egg production and economic returns. Known as the “King of Flavonoids,” *Ampelopsis grossedentata* is a green feed additive rich in flavonoid bioactive compounds, however, its effects on the reproductive performance of laying hens remain unclear. Therefore, this study investigated the impact of WEA on laying hens’ production performance, egg quality, reproductive function, and gut microbiota composition. Using metabolomics and network pharmacology approaches, we identified the primary active constituents of WEA and elucidated the potential mechanisms by which these constituents alleviate reproductive system aging. Furthermore, transcriptomic analysis combined with molecular docking techniques was employed to explore the underlying pathways through which WEA improves reproductive function and gut health. Additionally, we assessed WEA’s effects on apoptosis, inflammation, and antioxidant capacity in the ovary, oviduct, ileum and cecum, as well as its influence on the gut microbial community. The results demonstrated that dietary supplementation with WEA enhanced egg quality and reproductive organ health in laying hens, with these beneficial effects closely associated with improvements in gut microbiota structure and modulation of the PI3K/AKT signaling pathway and its downstream cascades. Collectively, these findings suggest that WEA represents a promising natural intervention for enhancing reproductive function in laying hens.

## Ethics approval and consent to participate

All experimental procedures involving animals were conducted in accordance with institutional and national guidelines for the care and use of laboratory animals. The study protocol was reviewed and approved by the Animal Ethics Committee of Hunan Agricultural University, under approval number [2022021].

## Declaration of generative AI and AI-assisted technologies in the writing process

During the preparation of this work the author(s) did not use any AI and AI-assisted technologies.

### Data availability

The datasets were deposited in a repository and are available at https://www.ncbi.nlm.nih.gov/sra/PRJNA1302706. Information can be made available from the authors upon request.

## CRediT authorship contribution statement

**Yu Xiao:** Writing – original draft, Visualization, Validation, Software, Resources, Methodology, Investigation, Data curation. **Jie Long:** Writing – original draft, Visualization, Validation, Software, Resources, Methodology, Investigation, Data curation. **Lu Liu:** Visualization, Validation, Resources, Methodology, Investigation, Formal analysis, Data curation. **Zhaojie Wang:** Visualization, Validation, Resources, Methodology, Investigation, Formal analysis, Data curation. **Wei Wang:** Resources, Methodology. **Peng Huang:** Writing – review & editing, Supervision, Project administration, Methodology, Funding acquisition, Conceptualization.

## Disclosures

The authors declare that they have no known competing financial interests or personal relationships that could have appeared to influence the work reported in this paper.

## Appendix Supplementary materials

Image, application 1

Image, application 2
