# Supplementary material for: Water extract of Ampelopsis grossedentata improves reproductive performance in laying hens by regulating gut microbiota and PI3K/AKT signaling pathway
Source: Poult Sci. 2025 Dec 31;105(3):106368. doi: 10.1016/j.psj.2025.106368 (PMC12809730; doi:10.1016/j.psj.2025.106368)
Supplement: Supplementary file 1 [file mmc1.docx]

**
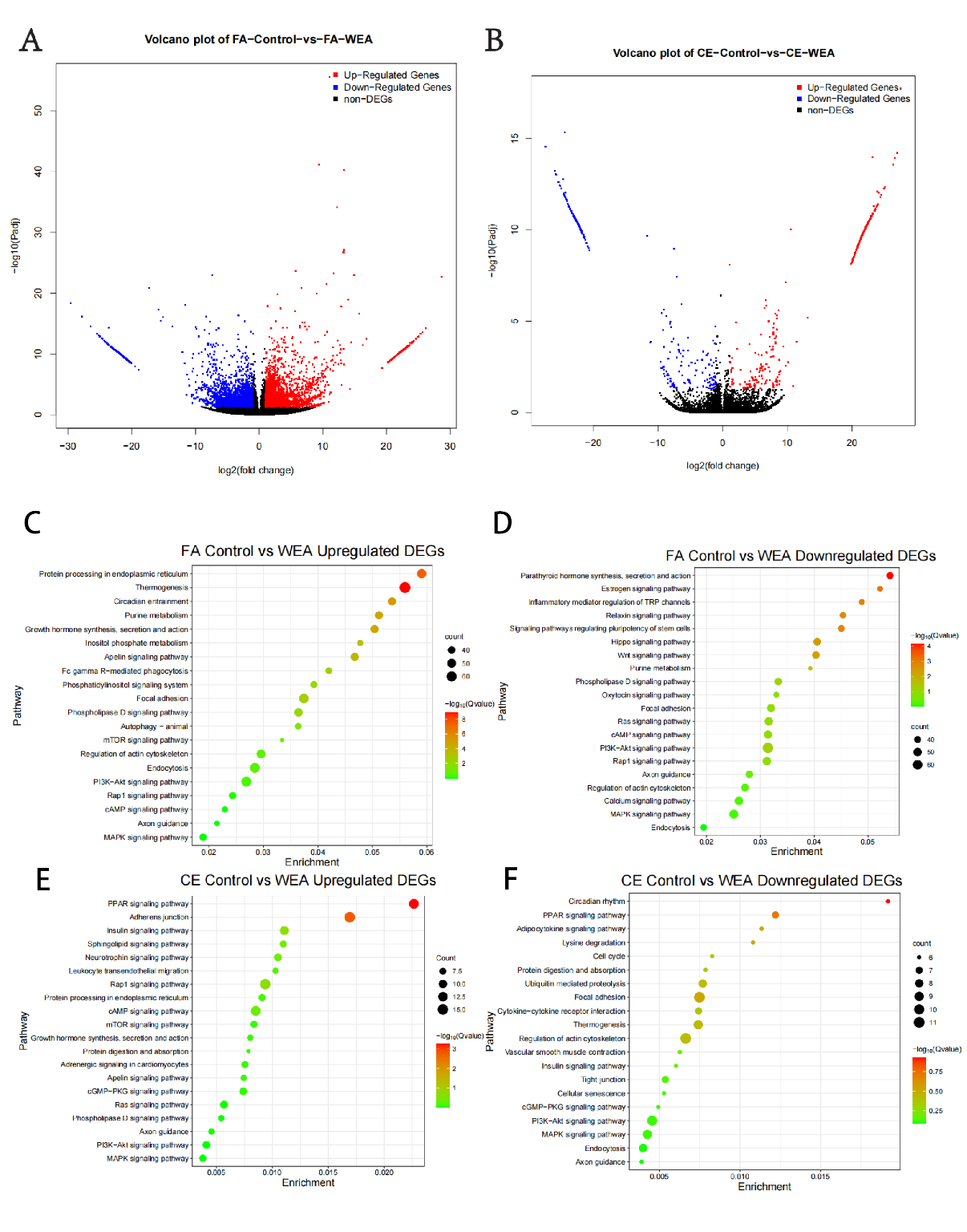
**

**Figure.4** Transcriptome and enrichment pathway analysis of samples. Volcano plot analysis of significantly different genes in (A) fallopian and (B)cecum in Control vs WEA. Significant DEGs were selected using the criteria of FC>=1 and P <0.05 in the volcano plot; Enrichment pathway analysis of(C-D) fallopian and(E-F) cecum. The degree of enrichment was analyzed by a rich factor, p-value, and the number of metabolites enriched in each pathway. The bubble size indicates the amount of significantly different species that are enriched in this pathway. The point with different gradations of color represents the scope of the p-value. The higher value of the rich factor stands for the higher degree of enrichment, and the lower p-value represents the extremely significant degree of enrichment.


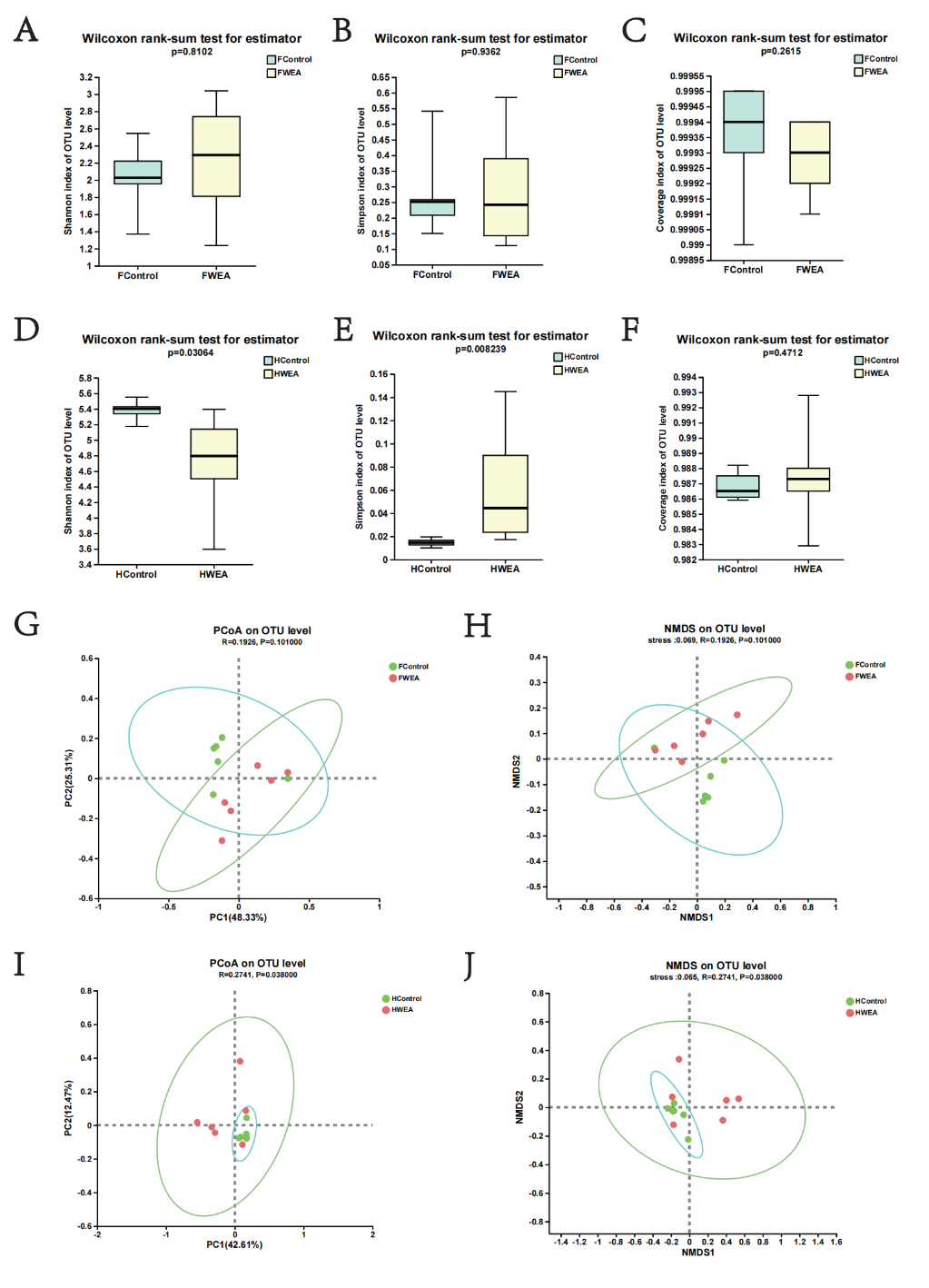


**Figure.s2** Bacterial alpha diversity and beta diversity analysis in the foregut (A, B, C, G, H) and hindgut (D, E, F, I, J).
