# Supplementary material for: Water extract of Ampelopsis grossedentata improves reproductive performance in laying hens by regulating gut microbiota and PI3K/AKT signaling pathway
Source: Poult Sci. 2025 Dec 31;105(3):106368. doi: 10.1016/j.psj.2025.106368 (PMC12809730; doi:10.1016/j.psj.2025.106368)
Supplement: Supplementary file 2 [file mmc2.docx]

Table s1.Ingredients and nutrient composition of the basal diet.

| Items | Composition(%) |
| --- | --- |
| Ingredients |  |
| Corn | 55 |
| Soybeanmeal | 25.1 |
| bran | 4 |
| Calciumhydrogenphosphate | 1.5 |
| Stonepowder | 9 |
| Coarsegrainstone | 2.7 |
| Premix1 | 3 |

Nutrient composition

| Metabolicenergy,MJ/kg | 11.48 |
| --- | --- |
| Crudeprotein,% | 15.773 |
| Crudefat,% | 2.56 |
| lysine,% | 0.76 |
| methionine,% | 0.35 |
| calcium,% | 3.65 |
| Totalphosphorus,% | 0.59 |
| effectivephosphorus,% | 0.37 |

^1^Premix feed provided per kilogram of complete feed: Fe 60mg, Cu 11 mg, I 0.4 mg, Zn 70mg, Mn 115 mg, Se 0.30 mg.

^1^Premix feed provided per kilogram of complete feed: VA, 6000 IU; VD3, 2500 IU; VE, 25.0 mg; VK3, 2.25 mg; VB1, 1.8 mg; VB2, 7.0 mg; VB6, 4.0 mg; VB12, 0.20 mg; pantothenic acid, 12.0 mg; niacin, 35.0 mg; biotin, 0.14 mg; folic acid, 0.8 mg. Nutrient levels are calculated values.

Table s2.The sequence of the object primers.

| Genes | Forward | Reverse |
| --- | --- | --- |
| β-actin | ATGATGATATTGCTGCGCTCGT | CCCATACCAACCATCACACCCT |
| ZO-1 | TATAGAAGATCGTGCGCCTCC | GAGGTCTGCCATCGTAGCTC |
| MUC2 | TTCATGATGCCTGCTCTTGTG | CCTGAGCCTTGGTACATTCTTGT |
| Occludin | CGGAGCCCAGACTACCAAAG | TTACACAGCTTCAGCCTTACA |
| Claudin-1 | GGTATGGCAACAGAGTGGCT | CAGCCAATGAAGAGGGCTGA |
| Nrf2 | CTGCCCAAAACTGCCGTA | CAAATCTTGCTCCAGTTCCA |
| HO-1 | GCTGAAGAAAATCGCCCAA | ATCTCAAGGGCATTCATTCGG |
| Keap1 | TGCCCCTGTGGTCAAAGTG | GGTTCGGTTACCGTCCTGC |
| NQO1 | GTTCAATGCCGTGCTCTCAC | CCGCTTCAATCTTCTTCTGC |
| IL-6 | CAACCTCAACCTGCCCAA | GGAGAGCTTCCTCAGGCATT |
| IL-10 | CACAACTTCTTCACCTGCGAG | CATGGCTTTGTAGATCCCGTTC |
| IL-4 | GTGCCCACGCTGTGCTTAC | AGGAAACCTCTCCCTGGATGTC |
| COX-2 | CTGCTCCCTCCCATGTCAGA | CACGTGAAGAATTCCGGTGTT |
| NF-κB | GATCTGCTGCCCCTGTACCTG | AGCTGAGCGCÇCTTCACACT |
| PTGEs | CTCAGCGTGGATTTCAACAACTGG | TCAGAAAAGCGATCAAAATTGGAC |
| TNF-α | TGTGTATGTGCAGCAACCCGTAGT | GGCATTGCAATTTGGACAGAAGT |
| PR | TGTTGTATTTTGCACCGGAT | GTGCTTTCATACATAGAAACTCCT |
| LHR | CTCAGGCGGATACACAACGA | TCAGAACGGCTTCCAGCAGG |
| E2R | CGTCCTAGCAAACCATTCACC | GCTTTCCAAGAGCCTGACT |
| Bcl-2 | ACCATGAATGAAACCGTGCC | TTGTCGTAGCCTCTTCTCCC |
| Bax | GTACGTCAATGTGGTCACCC | TGGGATAATGCTGGGGTTGA |
| Caspase3 | AAAGATGGACCACGCTCAGG | TGAACGAGATGACAGTCCGG |
| P53 | GCGCCGTGGCCGTCTATAA | GACTGCTGCGCCTCATTGATCT |
| Akt | GGCTACAAGGAACGACCGCAAG | TACTGTGGTCCACTGGAGGCATC |
| PI3K | TCGTGCAGCACAATGACTCT | CCTTCTCTCTCCAGAGGCCT |

Table s3. Main components and content of WEA

| Compounds | Class | WEA (nmol/g) |
| --- | --- | --- |
| Dihydromyricetin | Flavanonols | 3075.86 |
| Myricitrin | Flavonols | 470.66 |
| (-)-Catechin | Flavanols | 264.96 |
| Quercitrin | Flavonols | 250.63 |
| (-)-Epicatechin | Flavanols | 66.11 |
| (-)-Catechin gallate | Flavanols | 54.01 |

Table s4.Effects of dietary WEA on the performance of laying hens

|  | Mean±SEM | | | | P-value |
| --- | --- | --- | --- | --- | --- |
|  | Control | WEA-L | WEA-M | WEA-H |  |
| Laying rate,% | 91.63±0.73 | 91.67±0.92 | 89.86±0.59 | 91.43±0.75 | 0.286 |
| Egg weight,g | 61.54±0.64 | 62.96±0.25 | 62.53±0.43 | 61.70±0.20 | 0.098 |
| Misshapen eggs rate,% | 0.15±0.04 | 0.08±0.05 | 0.18±0.05 | 0.09±0.04 | 0.424 |
| Feed intake,g | 113.50±0.53 | 113.04±0.16 | 112.58±0.21 | 112.89±0.19 | 0.267 |
| Feed-egg ratio | 2.02±0.02 | 1.98±0.02 | 2.03±0.03 | 2.03±0.02 | 0.512 |

Control: basal diet; WEA-L: basal diet+50mg/kg Water extract of A. grossedentata; WEA-M: basal diet+150mg/kg Water extract of A. grossedentata; WEA-H: basal diet+250mg/kg Water extract of A. grossedentata; SEM: Standard error of the mean.

Table s5.Effects of dietary WEA on egg quality

|  | Mean±SEM | | | | P-value | |  |
| --- | --- | --- | --- | --- | --- | --- | --- |
|  | Control | WEA-L | WEA-M | WEA-H |  |  |  |
| Egg weight,g | 59.42±0.71^b^ | 65.77±1.57^a^ | 63.52±0.83^a^ | 64.90±0.56^a^ | | 0.001 | |
| Egg shape index,% | 73.24±0.60^c^ | 79.66±1.06^a^ | 77.75±0.57^ab^ | 77.11±0.46^b^ | | 0.000 | |
| Egg yolk color | 4.37±0.18^b^ | 5.00±0.19^a^ | 4.88±0.13^a^ | 4.88±0.13^a^ | | 0.044 | |
| Hastelloy units | 65.63±2.49 | 75.16±2.38 | 69.98±2.32 | 73.22±3.51 | | 0.096 | |
| Protein height,mm | 6.70±0.26^b^ | 7.94±0.29^a^ | 7.32±0.25^ab^ | 7.70±0.16^a^ | | 0.028 | |
| Egg shell strength,kgf | 3.00±0.14^b^ | 3.26±0.10^ab^ | 3.69±0.23^a^ | 3.45±0.10^ab^ | | 0.020 | |
| Egg shell thickness,mm | 0.29±0.01^b^ | 0.39±0.01^a^ | 0.39±0.03^a^ | 0.15±0.01^c^ | | 0.000 | |
| Egg yolk index | 0.35±0.01^b^ | 0.39±0.01^a^ | 0.39±0.01^a^ | 0.38±0.02^a^ | | 0.048 | |
| Egg yolk ratio | 0.28±0.00 | 0.29±0.01 | 0.29±0.00 | 0.29±0.01 | | 0.650 | |

^a,b^ Means within the same row not followed by the same letters are significantly different at *P* < 0.05. ^A, B, and C^ Means within the same row not followed by the same letters are significantly different at *P* < 0.01. Control: basal diet; WEA-L: basal diet+50mg/kg Water extract of A. grossedentata; WEA-M: basal diet+150mg/kg Water extract of A. grossedentata; WEA-H: basal diet+250mg/kg Water extract of A. grossedentata; SEM: Standard error of the mean.

Table s6.Effects of dietary WEA on organ index of laying hens

|  | Mean±SEM | | | | P-value |
| --- | --- | --- | --- | --- | --- |
|  | Control | WEA-L | WEA-M | WEA-H |  |
| Liver index,% | 1.53±0.08^c^ | 2.06±0.04^a^ | 1.75±0.05^b^ | 1.85±0.09^b^ | 0.000 |
| Spleen index,% | 0.07±0.00^b^ | 0.08±0.00^ab^ | 0.09±0.01^a^ | 0.09±0.01^a^ | 0.038 |
| Ovary index,% | 0.35±0.02 | 0.38±0.03 | 0.35±0.03 | 0.34±0.03 | 0.793 |
| Fallopian index,% | 3.13±0.14 | 3.63±0.15 | 3.05±0.13 | 3.28±0.15 | 0.076 |

^a,b^ Means within the same row not followed by the same letters are significantly different at *P* < 0.05. ^A, B, and C^ Means within the same row not followed by the same letters are significantly different at *P* < 0.01. Control: basal diet; WEA-L: basal diet+50mg/kg Water extract of A. grossedentata; WEA-M: basal diet+150mg/kg Water extract of A. grossedentata; WEA-H: basal diet+250mg/kg Water extract of A. grossedentata; SEM: Standard error of the mean.

Table s7.Molecular docking verification results

| Targetname | PDBID | Activeingredients | Bindingenergy(KJ/mol) |
| --- | --- | --- | --- |
| PI3K | 6HOG | (-)-Catechingallate | -7.014 |
|  |  | (-)-Catechin | -6.91 |
|  |  | (-)-Epicatechin | -6.929 |
|  |  | Myricitrin | -6.882 |
|  |  | Dihydromyricetin | -7.129 |
|  |  | Quercitrin | -6.796 |
